# The ConNECT approach: toward a comprehensive understanding of meaningful interpersonal moments in psychotherapy and beyond

**DOI:** 10.3389/fnhum.2025.1549203

**Published:** 2025-03-19

**Authors:** Niclas Kaiser, Juan Camilo Avendano-Diaz

**Affiliations:** ^1^Department of Psychology, Umeå University, Umeå, Sweden; ^2^Department of Neuroscience and Biomedical Engineering, Aalto University, Espoo, Finland

**Keywords:** psychotherapy, multi-person neuroscience, client-therapist interaction, interpersonal dynamics, subjective experience, convergence research, interpresence

## Abstract

Relational neuroscience struggles to capture the complex dynamics of shared interpersonal moments, leading to gaps in understanding whether and how interdependencies between interacting persons translate into something meaningful. Current neuroscientific research often focuses on motor synchronization and cognition rather than the implicit relational qualities central to psychotherapy. We argue that this disconnect stems from an over-reliance on simplified quantitative methods, a failure to centralize experiential factors, and the lack of Convergence research. Drawing on emerging frameworks such as 4E cognition (embodied, enacted, extended, and embedded) and MoBI (Mobile Brain/Body Imaging), we advocate for integrating subjective and experiential elements with neural data. We propose focusing on “qualities” in multi-brain neuroscience—moving beyond binary or linear scales—to better capture the subtleties of relational moments. Finally, we emphasize the importance of convergence research across disciplines to better understand what interpresence holds. If psychotherapeutic knowledge is used to guide neuroscientists in what to look for, this multi-disciplinary approach holds promise for advancing the study of psychotherapy’s relational processes, offering new insights into the neurobiology of meaningful moments in therapy and elsewhere. We propose ConNECT (Convergence research including Neuroscience and Experiences, Capturing meaningful dynamics with Therapists’ knowledge) as the path forward.

## 1 Introduction

Since its inception, neuroscience has generated a vast body of knowledge, providing invaluable insights into how the brain supports human cognition and behavior. Recent advancements have further expanded its boundaries by shifting research from highly controlled, lab-based settings to semi-naturalistic and naturalistic scenarios, where brain and body activity can be measured simultaneously in one or more individuals (Costa-Cordella et al., 2024; Hari et al., 2015; Stangl et al., 2023). These developments hold great potential for enhancing the ecological validity of research outcomes and deepening our understanding of brain function, brain-body interactions (Engelen et al., 2023; Parviainen et al., 2022), and their role in affective processes (Barrett, 2017). Moreover, they facilitate a more comprehensive investigation of relationships between interacting brains and bodies (Schilbach and Redcay, 2024), both in healthy individuals and in clinical contexts (Costa-Cordella et al., 2024; Crum, 2021). A particularly relevant domain where these advancements could converge is the study of meaningful shared interpersonal moments, with all their complexities and experiential phenomena.

The experience of meaningful shared interpersonal moments is crucial in psychotherapy and also of great importance in everyday life. Terms such as social connectedness (Kim and Sul, 2023), intersubjectivity (Fuchs and De Jaegher, 2009; Schore, 2021) social closeness (Egozi et al., 2022), mutuality (Cornelius-White et al., 2018; Murphy and Cramer, 2014) and shared moments (Stern, 2004) lie at the core of relational dynamics and are well-recognized in psychotherapy, particularly in approaches that focus on relational and psychodynamic processes. Research in psychotherapy has long acknowledged these relational processes as critical for therapeutic change, as seen in various models and interventions. For instance, the Boston Change Process Study Group (BCPSG) and their influential work on “moments of meeting,” emphasizing how authentic responsiveness can be used to facilitate therapeutic change (Stern et al., 1998). Without excluding other forms of psychotherapies, we highlight contemporary psychodynamic and relational psychotherapeutic approaches, e.g., the Accelerated Experiential Dynamic Psychotherapy (AEDP) model with its focus on the therapist’s active emotional participation (Goto et al., 2022; Markin et al., 2018). These models highlight the shared here and now as a therapeutic opportunity that is not unique for psychotherapy but represents a central human ability for shaping the sense of self and we-ness in close interaction with others (Kaiser and Butler, 2021; Stern, 2004). To contribute more effectively to understanding these relational processes, neuroscience would benefit from incorporating the unique experiences that take place in the shared present, drawing upon the extensive psychotherapeutic knowledge developed in this domain.

At present, a functional neural theory around shared interpersonal moments that corresponds to lived experience is needed, and “relational neuroscience” is the discipline closest at hand to address this. We refer to relational neuroscience as the “area of neuroscientific research that aims to model human sociality, with a specific focus on how people form, engage in, and maintain social relationships” (Felice et al., 2024, p. 2.). Many studies of relational psychology and neuroscience are based on self-reports and video observations, and several recent or ongoing projects are moving toward psychophysiological measures and brain imaging in dyadic settings (e.g., hyperscanning), with a focus on brain-to-brain coupling. It has been repeatedly reported that when individuals engage in social interaction, part of their neural activity across different timescales (e.g., at the level of brain rhythms or slower hemodynamic responses) becomes coupled or synchronized, a phenomenon known as inter-brain coupling or inter-brain synchrony. These findings span multiple tasks, including interpersonal coordination, joint action, cooperation, natural communication, music performance, and parent–child interactions (Czeszumski et al., 2020; Hari et al., 2013; Redcay and Schilbach, 2019; Turk et al., 2022; Zamm et al., 2024; Zhao et al., 2024). Such interdependencies between interacting brains are currently thought to reflect or even facilitate social interactions (Czeszumski et al., 2020).

The increasing body of inter-brain coupling literature has sparked some reactions, with several authors pointing out challenges and controversies in the field (Hakim et al., 2023; Nam et al., 2020; Novembre and Iannetti, 2021; Zamm et al., 2024). Researchers have questioned the lack of a solid theoretical framework (Holroyd, 2022), whether inter-brain coupling findings are genuinely informative of social interactions or merely epiphenomenal (Burgess, 2013; Hamilton, 2021; Holroyd, 2022), and whether they causally translate into social interaction (Novembre and Iannetti, 2021). In addition, the plethora of available methods and the lack of consensus on their application have also been criticized (Hakim et al., 2023; Holroyd, 2022). Moreover, these findings remain detached from the intricate relational processes at the heart of psychotherapy, and do not correspond well to the sense of what the dynamics of the interpersonal meeting holds in psychotherapy, the question of intersubjectivity as a mutually dynamic and clinically important process (Kaiser and Butler, 2021).

In many ways, the shared meaningful interpersonal moments in psychotherapy represent the ‘dark matter’ of relational neuroscience. Much like how Pfeiffer et al. (2013) describe the uncharted aspects of real-time social interaction, the nuanced and dynamic interpersonal exchanges that underpin therapeutic change remain elusive in current neuroscientific approaches, despite their centrality in psychotherapy and the extensive body of research on interpersonal autonomic physiology in therapeutic settings (e.g., Koole and Tschacher, 2016; Palumbo et al., 2017; Tschacher and Meier, 2019). Some initial attempts have been performed in the context of patient-clinician interaction in the treatment of chronic pain (e.g., Ellingsen et al., 2020) and psychological counseling (e.g., Zhang et al., 2018). Ellingsen et al. (2020) investigated inter-brain coupling between chronic pain patients and licensed acupuncturists using fMRI hyperscanning. Patients received experimentally induced pain, while clinicians provided pain relief through electroacupuncture needles triggered by a button press. During the anticipation of pain relief, dyads with a pre-established clinical relationship exhibited stronger inter-brain coupling in regions associated with social mirroring and theory of mind. Additionally, pre-stimulus coupling between the patient’s and clinician’s right temporoparietal junction (rTPJ) correlated negatively with the patient’s post-stimulus pain ratings, suggesting a potential link between inter-brain synchrony and pain modulation. However, due to the constraints of fMRI, the interactions were video-based and highly artificial, lacking the complexity of naturalistic exchanges and missing the moment-to-moment dynamics that may be crucial for meaningful therapeutic interactions. Similarly, Zhang et al. (2018) reported higher rTPJ synchronicity in client-counselor sessions compared to chat groups, which correlated with the post-conversation self-reported Working Alliance scores measured using the Working Alliance Inventory-Short Revised (WAI-SR; Munder et al., 2010). However, this was done without investigating the details of the dynamic interpersonal exchanges occurring during these sessions, which are proposed to be the central drivers of change. Furthermore, Akimoto et al. (2021) used fNIRS in sandplay therapy, finding correlations in the brain activity of therapists and clients in the frontopolar and prefrontal cortex during sandplay and post-therapy interviews, though without clear linkage to meaningful therapeutic processes. Notably, a synthesis of ideas bridging neurobiology and intersubjectivity has been proposed, suggesting, for instance, right-brain-to-right-brain synchronization as a potential model for understanding the key role of intersubjectivity in therapeutic change (Schore, 2022; Schore, 2021). However, a systematic literature review of the twelve studies to date on inter-brain coupling in clinical interactions suggests that the relationship between inter-brain dependencies and various aspects of the therapeutic process/relationship remains underexplored, and poorly understood (Adel et al., 2024).

Despite the development of widely accepted concepts such as therapeutic alliance (Wampold and Imel, 2015), attachment theory (Bowlby, 1973), the mirror-neuron system (Rizzolatti and Craighero, 2004), and emotion regulation theory (Gross, 2015), and other remarkable contributions within psychology and neuroscience, neuroscience continues to struggle with capturing the complex dynamics of relational change processes in psychotherapy research in a clinically relevant and useful manner. In addition, psychotherapy research has been criticized for relying too heavily on ‘mechanisms’ of therapeutic change defined in conceptual or statistical terms, which often fail to capture the precise functional bio-psychosocial foundations underlying this process (Carey et al., 2020).

We propose that the existing gap between relational neuroscience and interpersonal psychotherapeutic processes is partly due to three factors: (1) failure to centralize and operationalize experiential factors in psychotherapeutic change, highlighting the need for an integration of phenomenological research and related approaches. (2) Reliance on overly simplified quantitative concepts such as synchronicity, coupled with a tradition of employing methods that were originally developed for single-person neuroscience and (3) lack of knowledge sharing across disciplines such as psychotherapy, social neuroscience and philosophy of mind, calling mainly for the inclusion of psychotherapists knowledge in relational neuroscience-studies, but also for convergence research teams.

We propose ConNECT (Convergence research including Neuroscience and Experiences, Capturing meaningful dynamics with Therapists’ knowledge) as the path forward. ConNECT integrates three core elements essential for studies aiming to generate meaningful insights into the relational aspects of psychotherapy (and everyday life): (1) Inclusion of subjective/experiential dimensions, (2) Capturing meaningful relational dynamics from multi-person brain/body data using psychotherapists’ knowledge, and (3) Convergence research on interpresence, i.e., the shared meaningful here-and-now that enables unique relational processes not found elsewhere. The novelty and strength of our proposal lie in the integration of these elements. In addition, generating more meaningful results could offer valuable insights for clinicians in several ways. First, they could help develop objective measures for providing feedback, particularly in training therapists in relational skills. In a somewhat hypothetical future, psychotherapeutic sessions could benefit from integrating ConNECT with social biofeedback. Additionally, they would contribute to theoretical advancements by enabling the testing—and potential rejection—of hypotheses, thereby improving psychotherapy education programs, and the impact of psychotherapy itself. The CoNECT pathway could provide a deeper understanding of the impact of significant relational moments in psychotherapy, both shaping clinical processes and influencing long-term patient outcomes. Lastly, since intersubjective processes are fundamental (Kaiser and Butler, 2021), meaningful scientific results could also inform supportive interventions for individuals who struggle significantly with social interaction. While the core elements of ConNECT are applicable to any psychotherapeutic process and the study of meaningful interpersonal moments in everyday life, psychotherapeutic approaches that focus on relational and psychodynamic processes may offer a valuable starting point, given the priority they give to core relational moments.

**FIGURE 1:**
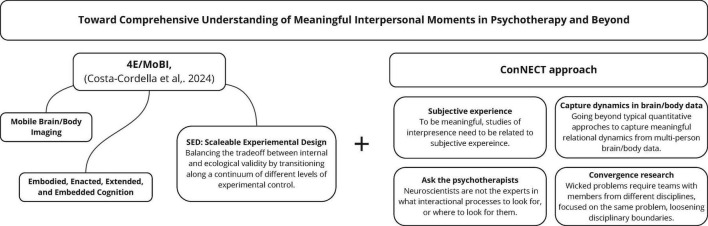
ConNECT approach as a framework for advancing the understanding of meaningful interpersonal moments in psychotherapy and beyond. Convergence research including Neuroscience and Experiences, Capturing meaningful dynamics with Therapists’ knowledge.

We provide details on each of the CoNECT core elements in the following sections. The ConNECT approach is summarized in Figure 1.

## 2 Inclusion of subjective and experiential dimensions

What is the relationship between the biological functions involved in nutrition intake and the experience of being hungry? Just as hunger may be an unreliable measure of the need to eat, it is the central experience driving feeding behavior. Similarly, subjective experiences of togetherness, though hard to measure, point to critical relational processes in therapy and serve as essential indicators of relational health (Kaiser and Butler, 2021). We propose that neuroscientific efforts to study dynamic interpersonal processes should aim to capture these strong experiences. Not necessarily as reliable measures of specific dimension, but by taking the phenomenological aspect of intersubjective experience seriously in the search for a framework that integrates neuroscience in meaningful ways outside the laboratory.

The lack of contact between neuroscience and subjective experience in psychotherapy seems to correspond to other areas as well. For example, in psychiatric research, the gap between subjective experience and neuroscience has been increasingly recognized, with growing calls to integrate phenomenology and neuroscience as a pathway to generating testable hypotheses about the biological basis of mental illness (Kyzar and Denfield, 2023). Integrating first-person methods, which focus on investigating psychological phenomena from the subjective, first-person perspective, is crucial for capturing the finer nuances of experience in therapeutic and relational contexts. Lumma and Weger (2023) provide a comprehensive overview and comparison of established first-person methods, including autoethnography, descriptive experience sampling, heuristic inquiry, micro-phenomenology, phenomenological approaches, systematic introspection, and thinking aloud. Many of these methods have strong potential for successful incorporation into the neuroscientific study of meaningful interpersonal moments in psychotherapy and beyond. For instance, phenomenological approaches have already been applied in studying subjective experiences during meditative states (Nave et al., 2021), epilepsy (Le Van Quyen and Petitmengin, 2002), awareness during sleep (Alcaraz-Sánchez et al., 2022), chronic pain (Smrdu, 2022; Valenzuela-Moguillansky, 2013), empathy for pain (Martínez-Pernía et al., 2023) and the understanding of consciousness (Jo et al., 2014; Timmermann et al., 2023; Varela, 1996), as well as in research on the neural dynamics modulated by subjective experiences under psychedelic states (Lewis-Healey et al., 2024; Timmermann et al., 2023) and psychedelic-assisted psychotherapy (Ventura, 2024). Part of our invitation is to look into these research branches for inspiration. Incorporating these methodological approaches into the study of relational moments in psychotherapy constitutes a promising avenue. Further details/suggestions on how subjective and experiential dimensions can be used to guide research within CoNECT are provided in section 3.

These experiences could then be linked to physiological and neural markers accompanying them. For instance, in contexts such as dance improvisation, moments of togetherness have been described as states of “being in the zone,” where participants experience a dissolution of self-other boundaries and a shared, unselfconscious awareness (Noy et al., 2015). These subjective experiences, while difficult to quantify, are marked by physiological indicators such as heart rate synchronization and heightened engagement (Noy et al., 2015) and are still awaiting multi-brain markers that could further enhance our understanding.

## 3 Capturing meaningful relational dynamics from multi-person brain/body data, using psychotherapists’ knowledge

The way relational neuroscience studies are set up—through their research questions, experimental settings, analysis methods, and statistical measurements—shapes the resulting discussions and interpretations. For example, reliance on dichotomous variables (e.g., “it is there or not”), singular terms like “it” (e.g., connection), and scales of “more or less synchronicity” have made it challenging to connect science with subjective experience in general and in psychotherapy specifically. We propose that such concepts risk deepening the gap between experience and neuroscience, and hinder efforts to cross disciplinary boundaries. These apply to both classic single-brain neuroscience studies, and more recent multi-brain hyperscanning research. While we acknowledge that multi-brain/body data has significant potential to revolutionize relational neuroscience (Hari et al., 2015; Hasson et al., 2012), we consider that hyperscanning studies should expand their focus beyond inter-brain synchrony perspectives (Friston and Frith, 2015; Froese et al., 2024; Laroche et al., 2024; Li et al., 2025; Sarasso et al., 2024). For instance, this could involve developing and incorporating innovative computational methods that transcend traditional synchrony analysis, e.g., two-brain microstates to quantify inter-brain asymmetries (Li et al., 2025), or considering and testing complementary hypotheses that might broaden our understanding of interacting brains and bodies. One such example is the irruption theory, which suggests that increased subjective involvement in social interactions might lead to heightened neural entropy and inter-brain desynchronization (Froese et al., 2024). This aligns with the concept of the “problematization of coordination,” where disrupting synchrony serves as an active strategy for fostering creative change (Laroche et al., 2024), and with emerging research emphasizing the role of disorganization, desynchronization and entropy as key markers of therapeutic change (Sarasso et al., 2024).

Instead of relying on conventional cognitive neuroscience to study processes in psychotherapy, we propose reversing the approach: using clinical expertise to guide neuroscience by identifying what to look for and where to look for it, focusing on clinically meaningful interpersonal moments. There are several reasons to use psychotherapy as a window to relational neuroscience: (1) Evidence shows that the quality of the patient-therapist processes is closely associated with therapeutic change, mirroring the impact of positive relationships in everyday life. (2) Psychotherapy offers an abundance of clinically proven experiences from trained therapists and their patients, as well as a vast body of theoretical development on change processes. (3) Psychotherapy takes place in a semi-controlled environment, and employs semi-controlled methods, balancing ecological validity and experimental control. (4) There is a long tradition in psychotherapy of working toward explicit goals, while understanding their complexity and multifaceted nature.

Parts of what we argue for have been proposed by Costa-Cordella et al. (2024) with 4E/MoBI as a path for advancing the neuroscience of psychotherapy. 4E points to studying multiple modalities of the human system, i.e., embodied, enacted, extended, and embedded. MoBI stands for Mobile Brain/Body Imaging, which integrates multiple data streams from dynamic brain and body measurements in real-world contexts. Additionally, Costa-Cordella et al. (2024) also proposed the use of Scaleable Experimental Design (SED), which emphasizes balancing internal and ecological validity by transitioning along a continuum of different levels of experimental control: starting with controlled experiments and then moving to semi-controlled and uncontrolled experiments. This approach also demands a clear paradigm to identify what phenomenon to look for, and where to look for it. Following this same principle of balancing ecological validity with experimental control, we propose e.g., using Moment-to-to-Moment Tracking (MtMTr), a method where the therapist follows patients’ experiences in the room, including their perceptions of the psychotherapist. This method is used in AEDP-model based therapies (Fosha, 2018) and related training workshops. We identify several advantages with MtMTr. It is typically time-limited and can be adapted to last for approximately 15 min in semi-controlled settings of therapist-therapist interaction, which has strong ethical advantages as it does not have to involve patients who are in the room for more important reasons than research. A recent pilot study of AEDP workshops have shown that therapists can deliberately enter and exit meaningful relational moments at will in therapist-therapist dyads, while wearing brain imaging equipment (Kaiser et al., 2025), allowing for multiple comparable trials, making it a promising candidate for use in an SED-setting, focusing on relational dynamics that matter.

An additional step forward would be to go beyond traditional quantitative analysis, integrating multi-person physiological data (brain and body) with phenomenological aspects of experience to uncover meaningful relational dynamics. One related approach is the protocol proposed by Tucek et al. (2022), which uses an anthropological research paradigm in combination with EEG/ECG hyperscanning, qualitative interviews, and video analysis to better understand the relational dynamics between therapists and stroke patients during music therapy sessions. In this protocol, after the therapy session, both the therapist and patient are asked separately to watch video recordings of their interactions and identify moments of interest (MOIs) and moments of non-interest (MONIs). This is complemented by qualitative interviews focusing on their reflections on the identified moments. From this data, a comprehensive profile of the MOIs is built, and the time series from the recorded neurophysiological data are then aligned with the MOI profile to examine their relationships. This process is closely related to other contemporary approaches like Temporal experience tracing (Jachs, 2022; Lewis-Healey et al., 2024). These integrative approaches provide an example of how multi-person neural relational qualities could be better understood by combining neuroscientific tools with qualitative insights. Notably, a related strategy was proposed by Costa-Cordella et al. (2024), termed “opportunistic sampling,” in which video analysis is used to create “natural trials” based on hypothesis-driven behavioral moments. Similarly, the use of natural language processing (NLP), AI-raters, and content analysis to identify key subjective and linguistic features of therapy could be a valuable complementary approach (Adel et al., 2024).

Bridging with the arts and related disciplines may offer additional insights into meaningful relational dynamics from multi-person brain/body data. In this context, we highlight Suzanne Dikkers’ work (Dikker et al., 2019), connecting artistic exploration with big data, scientific inquiry and tech-based communication tools as a potential path toward understanding experiences of feeling connected to one another. Her team has also shown how such naturalistic approaches can be applied in public spaces (Dikker et al., 2021). An additional example comes from research through design, a discipline that emphasizes how practice-based research and design practice are well-suited to generate new knowledge (Redström, 2017). This approach involves designing systems and experiences to investigate specific questions or hypotheses, in this case, hypotheses about the dynamic complexity of interpersonal processes in a shared here-and-now. One example is the work of Trotto et al. (2016), on dyads dancing in a MoCap Tango, where the interpersonal dynamics are visualized as shared movement over time, frozen in an image or a sculpture. This visualization captures the relational qualities embedded in the physical bodily dialogue between dancers, offering qualitative insights into the processes that unfolded during the interaction.

## 4 Convergence on interpresence

We identify bridging the gaps between experience, psychotherapy and neuroscience as a wicked problem (Rittel and Webber, 1973), fitting the criteria for wicked problems as it undoubtedly involves a high degree of complexity and uncertainty. Addressing this wicked problem requires pushing beyond current limits with help of convergence research where researchers from various fields focus on the same problem, loosening their disciplinary boundaries (Hari, 2023). The wicked problem of interest might be the nature of the “shared moment” and what it entails.

To support this interdisciplinary focus, we introduce the concept of “interpresence,” which we find helpful in describing the condition of being in a shared psychological here and now. We define “interpresence” as being together, at the same time, in the same psychological space—not necessarily the same physical space. The term is related to co-presence (Campos-Castillo and Hitlin, 2013; Goffman, 1963), which refers more directly to being in the same space. Interpresence focuses more on the “inter” aspect and requires an additional layer of mutual awareness and engagement, where both individuals actively engage with each other’s presence and mental states. In this sense, interpresence involves more than joint attention (Baron-Cohen, 1989), which focuses on an external shared focus, or mutual awareness (Soboroff et al., 2020), which simply involves recognizing another’s mental state. Instead, it serves as the basic condition for immersive, co-created experience of presence, where both parties co-experience the present moment in a shared psychological space. We find the term interpresence well suited for convergence, centering on entangled co-realities in dyads and small groups, as well as experiences of being entangled with embodied and disembodied AI (Hellström et al., 2024). We propose that it is in interpresence that unique moments of genuine connection can take place, where we can see and feel seen, talk and feel heard, affirm and feel validated-together.

## 5 Limitations

### 5.1 Patient’s needs and ethical concerns

In psychotherapy research, a key factor is the patient’s need. The therapeutic process is primarily designed to benefit the patient rather than serve scientific purposes. As a result, studying essential relational moments depends on the patient’s immediate needs, which may lead to a situation where very few, if any, significant moments are available for analysis. Furthermore, attempting to induce these moments raises ethical concerns, as doing so could interfere with a highly sensitive clinical process.

Another challenge is the laboratory setting and the use of brain imaging equipment, which are rarely designed to directly assist the individual patient. These setups often induce discomfort, the preparation process is time-consuming, and participants may feel self-conscious wearing an EEG or fNIRS cap. Additionally, the overall experience can feel detached from typical therapeutic settings. However, the twelve multi-person neuroscience studies conducted in therapeutic settings to date suggest that using neuroimaging equipment does not compromise the therapeutic process or the patient’s experience (Adel et al., 2024).

### 5.2 CoNECT and the signal-to-noise ratio (SNR) monster

When measuring brain activity, neural signals of interest are often small compared to other bodily signals and environmental noise, leading to a low signal-to-noise ratio (SNR). Improving SNR is critical for enhancing the accuracy, reliability, and interpretability of neuroimaging data. Traditional neuroimaging research compensates for this by, for instance, repeatedly presenting the same controlled stimulus and averaging multiple responses to enhance signals of interest.

However, naturalistic neuroscience often lacks the controlled settings, structured trial repetitions, and predefined event markers found in traditional experiments. When combined with real-world movement artifacts, increased environmental variability, and the complexity of multi-person interactions, these factors increase noise sources and further exacerbate SNR challenges.

At first glance, the CoNECT approach may seem like a nightmare for neuroscientists due to these challenges. However, while CoNECT does not provide the same level of experimental control as traditional cognitive neuroscience paradigms, it incorporates strategies to mitigate SNR limitations and enhance the reliability of acquired neural data (details on these approaches are presented in section 3), while offering alternative methodological pathways that prioritize ecological validity and clinical relevance. This is highly relevant, given that SNR challenges have been one of the main barriers preventing scientists from producing more meaningful results regarding the strong relational experiences that are believed to impact both clinical processes and everyday life.

Among the CoNECT strategies, Scalable Experimental Design (SED) allows for a structured transition from highly controlled experiments to semi-controlled and naturalistic settings while maintaining methodological rigor (Costa-Cordella et al., 2024). Combining controlled lab settings with naturalistic interactions can help balance ecological validity and SNR. We also propose a selective analysis approach, in which first-person methods, video analysis, therapist knowledge, and insights from artistic and design-based research are combined to identify key moments of interest in psychotherapeutic sessions (see also Tucek et al., 2022; Costa-Cordella et al., 2024; Adel et al., 2024; Dikker et al., 2019, 2021; Redström, 2017; Trotto et al., 2016). These moments are rigorously described and grouped based on specific criteria, allowing for a more structured aggregation of similar events, thereby increasing the number of analyzable trials. While this approach does not achieve the same experimental precision as classical paradigms, it aligns neuroscientific data with subjective experience and therapist expertise, potentially making it more clinically meaningful. Moreover, since therapy unfolds over multiple sessions, repeated measurements across sessions could further increase the number of useful trials and enhance statistical power, while also providing a more ecologically valid measure of the relational change process (Adel et al., 2024). Interestingly, in AEDP and related approaches, therapists can deliberately enter and exit meaningful relational moments at will in laboratory settings (Hanakawa, 2021; Kaiser et al., 2025), allowing for multiple comparable trials—a feature that could be leveraged to improve SNR.

Finally, we encourage researchers to follow the most up-to-date guidelines for their chosen neuroscience technique to ensure high-quality data collection. We also recommend following the developments in the Mobile Brain/Body Imaging field, as new algorithms and approaches continue to improve noise reduction and data quality in naturalistic neuroscience settings. Similarly, we strongly recommend following the latest guidelines on hyperscanning research (e.g., Zamm et al., 2024), as they provide essential recommendations for optimizing data quality and therefore, improving the SNR.

By implementing these strategies, CoNECT presents a viable path forward for studying meaningful interpersonal moments in psychotherapy and beyond.

## 6 Conclusion

In conclusion, the gap between relational neuroscience and psychotherapy research presents a critical challenge in understanding the neural basis of meaningful relational change processes. While shared interpersonal moments, social connectedness and intersubjectivity are central to psychotherapy, neuroscience has yet to fully explore the nuances and complexities of these experiential phenomena. We argue that the reliance on simplified models of social interaction and the lack of integration between neuroscience and psychotherapy limits progress. To address these challenges, we propose the ConNECT approach, which emphasizes including subjective and experiential dimensions, capturing meaningful relational dynamics from multi-person brain/body data, and embracing convergence research and therapists’ knowledge, as well as incorporating frameworks such as Scalable Experimental Protocols, and 4E/MoBI. This framework leverages psychotherapeutic knowledge to guide neuroscience in identifying where to look for biological underpinnings that can better capture the complexities of relational dynamics and foster meaningful advances in both relational neuroscience and psychotherapy. Central to this integrative approach is the concept of “interpresence,” which describes the shared psychological here and now that enables unique processes of mutual engagement and connection. We emphasize ConNECT interpresence as a path for convergence, capable of bridging disciplinary boundaries and advancing our understanding of the meaningful relational dynamics that underlie both psychotherapy and human connection.

## Data Availability

The original contributions presented in this study are included in this article/supplementary material, further inquiries can be directed to the corresponding author.
